# Ski-geometric parameters do not differ between ACL injury mechanisms in recreational alpine skiing

**DOI:** 10.1007/s00167-021-06852-w

**Published:** 2021-12-31

**Authors:** Markus Posch, Gerhard Ruedl, Klaus Greier, Martin Faulhaber, Katja Tecklenburg, Alois Schranz, Martin Burtscher

**Affiliations:** 1 Department of Sport Science of the University of Innsbruck, Fürstenweg 185, 6020 Innsbruck, Austria; 2 University College of Education (KPH) Stams, 6422 Stams, Austria; 3 medalp sportclinic, 6460 Imst, Austria

**Keywords:** ACL injury mechanisms, Circumstances of fall, Ski geometry, Recreational alpine skiing

## Abstract

**Purpose:**

It is not known so far if ski-equipment-related factors differ between the ACL injury mechanisms, potentially influencing the circumstances and causes of falling, finally resulting in ACL injury. More specifically focusing on the injury mechanisms will provide a deeper understanding of injury causation. The aim of the study was to evaluate whether ACL injury mechanisms in recreational alpine skiing differ with regard to ski-geometric parameters, self-reported circumstances and causes of accident and injury severity.

**Methods:**

Among a cohort of 392 ACL-injured (57.9% females) skiers, age, sex, height, weight, skill level, risk-taking behavior, circumstances and causes of accident, and ACL injury severity were collected by questionnaire. Additionally, patients had to recall their type of fall (ACL injury mechanism) by classifying forward and backward falls with and without body rotation. Ski length, side cut radius and widths of the tip, waist and tail were directly notated from the ski.

**Results:**

The forward fall with body rotation was the most common reported ACL injury mechanism (63%). A riskier behavior was associated with forward falls without body rotation. Ski-geometric parameters did not significantly influence the type of ACL injury mechanism. Regarding accident characteristics, catching an edge of the ski was more frequent (*p* < 0.001) the cause for forward falls (75% and 67%) when compared to the backward falls (46 and 15%) and executing a turn was the most frequent action in all falls (39–68%). A complete rupture of the ACL (66–70%) was more commonly reported than a partial tear (30–34%) among all four non-contact ACL injury mechanisms (n.s.).

**Conclusion:**

In contrast to risk-taking behavior and accident characteristics, ski-geometric parameters and injury severity do not significantly differ between ACL injury mechanisms in recreational skiing. Thus, an individual skiing style seems to have more impact on ACL injury mechanisms than ski equipment. Future studies should evaluate potential effects of ski geometry on the incidence of ACL injury.

**Level of evidence:**

III.

## Introduction

Recreational alpine skiing represents the most popular winter sport, annually enjoyed by several millions of people worldwide [5, 22]. Importantly, related to the enormous number of skiers practicing this sport is also associated with a certain risk for injury [7, 9]. Thus, thousands of ski injuries are recorded each year, e.g., in Austria, despite the fact that the injury rate is even less than one injury per 1000 skier days [20].

The knee joint represents the most commonly affected anatomical location accounting for about one-third of all injuries among female and male skiers [3, 6, 20]. A study by Majewski et al. [11] showed that almost 50% of knee-injured skiers ruptured their anterior cruciate ligament (ACL). Recently, Posch et al. [15] demonstrated, that ACL ruptures with concomitant injuries, mostly to the medial collateral ligament (MCL), occur in 65% of knee injuries, especially within males [26].

It is important to note, that female recreational skiers have twice the knee injury incidence of male skiers and the ACL injury risk is three times greater in females [2, 3, 6]. This fact may be also attributed to different hormonal, anatomical and neuromuscular risk factors between sexes [8].

Despite the complex interactions between intrinsic (age, sex, skill level, risk-taking behavior etc.) and extrinsic (snow-, and weather conditions, ski-boot-binding unit) risk factors associated with a skiing-related ACL injury [1, 4, 18], more specifically focusing on the injury mechanisms will provide a deeper understanding of injury causation. According to Natri et al. [12], four main non-contact ACL injury mechanisms in recreational skiing have been identified: (1) forward fall with body rotation (“valgus external rotation”), (2) backward fall with body rotation (“flexion internal rotation—phantom foot”) and (3) backward fall without body rotation “boot-induced anterior drawer” and (4) forward fall without body rotation.

Several epidemiological studies [15, 17–19, 21, 25] found that, since the introduction of the short and shaped carving skis in the late 90 s, the forward fall with body rotation was the most commonly reported injury mechanism accounting for about 49–69% among recreational alpine skiers suffering from knee injuries.

Up to date, there is only one study by Ruedl et al. [17], investigating the distribution of ACL injury mechanisms and comparing related risk factors between the forward twisting falls and others, however, among female carving skiers only. In addition, due to the small sample size all four ACL injury mechanisms could not be evaluated separately [17]. Another study by Ruedl et al. [18] indicated significant differences between male and female skiers with regard to circumstances of fall and actions when ACL injury occurred although the forward twisting fall with body rotation was the most common reported ACL injury mechanism in both sexes. However, this study [18] did not provide a comparison of accident characteristics with regard to different types of ACL injury mechanism.

Moreover, it is not known so far if ski-equipment-related factors as side cut radius and ski dimensions (widths of the tip, waist and tail) differ between the ACL injury mechanisms, potentially influencing the circumstances and causes of falling, finally resulting in ACL injury. A link between a greater ski length and a higher risk has been already reported for sustaining an ACL injury [10, 12]. Furthermore, it is known that skis with sportier settings that have a small waist width and a strong side cut are less forgiving [23]. As the four main ACL injury mechanisms are differentiated with regard to the injury sequences of the involved anatomical body parts [12, 17], it seems also of interest, whether the severity of the ACL injury varies between the injury mechanisms. It would be important to know, whether ski geometry influences ACL injury mechanisms and related injury severity.

It was hypothesized that ski-geometric parameters and accident characteristics would differ between ACL injury mechanisms. Therefore, the aim of the present study was to evaluate whether ACL injury mechanisms in recreational alpine skiing differ with regard to ski-geometric parameters, self-reported circumstances and causes of accident, and injury severity.

## Materials and methods

This study was conducted as a retrospective questionnaire-based survey of ACL-injured recreational skiers during six consecutive winter seasons (2014/15–2019/20), treated in a large sports clinic.

All study participants were informed about the aims of the study and gave their written informed consent for participating. The survey was conducted according to the ethical guidelines for surveys approved by the Institutional Review Board (IRB) as well as the Board for Ethical Issues (BfEI) of the University of Innsbruck (ID: 25/2016). Patients or the public were not involved in the design, or conduct, or reporting, or dissemination plans of this research.

In accordance to previous studies by Posch et al. [14, 15], cases were interviewed during their stay at hospital between the months December and April on 19–25 days (23 days on average) per winter season using a predefined standardized questionnaire. As described previously by Ruedl et al. [18] and Posch et al. [15], the random recruitment of patients was dependent upon logistical aspects at the sports clinic (availability of rooms and personnel) and willingness of patients to volunteer. In total, more than 95% of invited patients agreed to participate.

Inclusion criteria were a skiing-related non-contact ACL injury after a self-inflicted fall, an age > 17 years and the use of any type of carving skis (in contrast to long and unshaped traditional skis as well as to so-called short ski boards). ACL injuries were diagnosed by a physician using magnetic resonance imaging (MRI), which is directly available at the sports clinic [15]. Furthermore, regarding injury severity, injuries to the ACL were rated by the physician as partial (grade II) or complete tear (grade III) [15]. Grade II injuries of the ACL generate a perceived instability of the knee joint caused by partially torn fibers of the ligament. If the fibers of the ligament are completely torn and the ligament itself is torn in two parts leading to a loss of knee movement control the ACL injury was rated as grade III [15].

Depending on several factors like the severity of ACL injury, the number, type, and severity of concomitant knee injuries, different surgery techniques have been considered [13, 16]. In case of a high-grade partial or subtotal tear of the ACL, an acute biologic approach was used to preserve the native ACL. Healing response technique according to Steadman et al. [27] and ligament bracing techniques have been applied. Autograft ACL reconstruction was considered for complete tears [13]. Graft choice was made dependent on age, gender and sports activity level. Concomitant injuries (i.e. meniscus, cartilage, grade III tear of the medial collateral ligament) were additionally addressed. Conservative treatment and possible delayed ACL reconstruction were furthermore discussed with each patient and applied according to patient-related parameters such as low level of sports activity or for personal reasons.

In addition to anthropometric data (age, sex, height, weight), skiers were asked to self-report their risk-taking behavior (more risky vs. more cautious) according to Ruedl et al. [19] and skiing skill level (expert, advanced, intermediate and beginner) according to Sulheim et al. [28]. Furthermore, patients were divided into more skilled (expert and advanced) and into less skilled (intermediate and beginner) skiers as it was suggested to underestimate individual skiing skills, especially among female skiers [28].

Importantly, patients had to recall their type of fall by watching pictures of the most common ACL injury mechanisms in recreational alpine skiing. During the interviews, there was sufficient time to discuss the ACL injury mechanism and to recall the injury situation as best as possible. There were four different types of fall to choose: (1) forward fall with body rotation (valgus external rotation), (2) forward fall without body rotation, (3) backward fall with body rotation (flexion internal rotation—phantom foot) and (4) backward fall without body rotation (boot-induced anterior drawer). Moreover, with regard to accident characteristics also circumstances of the fall were asked and further divided into: (1) after jumping, (2) caught an edge, (3) ski slid out or away/lost balance, (4) accidental binding release and (5) others. Additionally, patients were asked for a potential cause whether the ACL injury occurred while (1) executing a turn, (2) going straight, (3) landing after jump and (4) do not know.

Ski dimensions like tip, waist and tail width, absolute ski length and side cut radius were directly notated from the ski. Additionally, ski length was relativized by body height to enable further analysis.

### Statistical analysis

No sample size calculation was conducted, but the goal was to have at least 150 ACL-injured skiers of both sexes. All data are presented as means, absolute and relative frequencies. In a first step, differences in frequencies (injury mechanisms, skiing skill level, risk-taking behavior, circumstances of fall, actions when ACL injury occurred, ACL injury severity) between all ACL injury mechanisms were evaluated by Chi-square tests. Univariate differences among metric data (age, height, weight, ski length relativized to body height, side cut radius and ski dimensions) between the four ACL injury mechanisms were evaluated by one-way ANOVAS. SPSS 24.0 (IBM Corporation, Armonk, NY) was used for the statistical analysis. All *p* values were two tailed and statistical differences were considered significant at *p* < 0.05.

## Results

A total of 392 recreational alpine skiers (57.9% females) suffering from a non-contact ACL injury with a mean age of 42.7 ± 10.4 years, mean height of 1.72 ± 0.1 m and a mean weight of 71.1 ± 11.2 kg volunteered for this study.

In general, the forward fall with body rotation (63%) was the most common self-reported ACL injury mechanism, followed by the backward fall with body rotation (25%), forward fall without body rotation (9%) and backward fall without body rotation (3%). With regard to a sex-specific comparison (Table 1), no significant difference was found within the distribution of ACL injury mechanisms. Both, females and males, reported the forward fall with body rotation being the dominant ACL injury mechanism with more than 60%.

**Table 1:** Differences of the distribution of injury mechanisms in ACL-injured recreational alpine skiers

	males [*n* = 165]	Females [*n* = 227]	*p* value
Injury mechanism [*n*, %]			n.s
Forward fall with body rotation	102 (61.9)	145 (63.9)	
Forward fall without body rotation	18 (10.9)	18 (7.9)	
Backward fall with body rotation	39 (23.6)	57 (25.1)	
Backward fall without body rotation	6 (3.6)	7 (3.1)	

Data are presented as absolute and relative frequencies

In Table 2, differences of the distribution of intrinsic risk factors, ski-geometric parameters, circumstances, causes and severity of ACL injury in recreational alpine skiers are represented.

**Table 2:** Differences of the distribution of intrinsic risk factors, ski-geometric parameters, circumstances, causes and severity of ACL injury in recreational alpine skiers

	Non-contact ACL injury mechanisms				
	Forward fall with body rotation [*n* = 247]	Forward fall without body rotation [*n* = 36]	Backward fall with body rotation [*n* = 96]	Backward fall without body rotation [*n* = 13]	*p* value
Intrinsic risk factors					
Age [years]	41.9 ± 10.4	42.8 ± 11.0	44.7 ± 10.3	42.5 ± 10.2	n.s
Height [m]	1.72 ± 0.08	1.72 ± 0.08	1.73 ± 0.07	1.72 ± 0.08	n.s
Weight [kg]	70.6 ± 11.1	71.3 ± 10.2	72.7 ± 11.4	69.2 ± 13.9	n.s
Skiing skill level [*n*, %]					n.s
More skilled	133 (53.8)	18 (50.0)	46 (47.9)	9 (69.2)	
Less skilled	114 (46.2)	18 (50.0)	50 (52.1)	4 (30.8)	
Risk-taking behavior [*n*, %]					0.040
More risky	89 (36.0)	22 (61.1)	37 (38.5)	5 (38.5)	
More cautious	158 (64.0)	14 (38.9)	59 (61.5)	8 (61.5)	
Ski-geometric parameters					
Relativized ski length to body height [%]: mean ± SD	94.6 ± 4.0	96.9 ± 4.5	94.7 ± 4.3	95.0 ± 3.7	n.s
Sidecut radius [m]: mean ± SD	13.9 ± 2.1	14.2 ± 1.5	14.1 ± 2.0	14.8 ± 3.0	n.s
Ski dimension [mm]: mean ± SD					
Tip width	119.9 ± 6.5	122.5 ± 4.2	121.0 ± 7.2	120.8 ± 6.8	n.s
Waist width	72.5 ± 4.9	73.7 ± 3.5	73.7 ± 4.5	73.6 ± 4.0	n.s
Tail width	104.0 ± 6.2	103.9 ± 8.1	106.6 ± 8.3	105.5 ± 6.9	n.s
Accident characteristics					
Circumstances of fall [*n*, %]					< 0.001
After jumping	7 (2.8)	2 (5.6)	0	1 (7.7)	
Caught an edge	184 (74.5)	24 (66.7)	44 (45.8)	2 (15.4)	
Ski slid out or away/lost balance	53 (21.5)	10 (27.7)	52 (54.2)	10 (76.9)	
Inadvertent binding release	3 (1.2)	0	0	0	
Cause of accident [*n*, %]					0.007
Executing a turn	139 (56.3)	22 (61.2)	65 (67.7)	5 (38.5)	
Going straight	13 (5.3)	7 (19.4)	5 (5.2)	3 (23.1)	
Landing after jump	3 (1.2)	0	0	0	
Do not know	92 (37.2)	7 (19.4)	26 (27.1)	5 (38.5)	
ACL injury severity [*n*, %]					n.s
Partial tear (grade II)	73 (29.6)	11 (30.6)	33 (34.4)	4 (30.8)	
Complete rupture (grade III)	174 (70.4)	25 (69.4)	63 (65.6)	9 (69.2)	

Data are presented as mean ± SD, absolute and relative frequencies

### Intrinsic risk factors

Represented in Table 2, age, height, weight and skiing skill level of participants was not significantly different between the four ACL injury mechanisms. Risk-taking behavior (*p* = 0.040) significantly differs between ACL injury mechanisms. Patients suffering from a forward fall without body rotation most commonly reported to be more risky (61%), whereas patients who injured their ACL due to the other three ACL injury mechanisms reported to be more cautious (62–64%).

### Ski-geometric parameters

Comparing ski-geometric parameters between the four ACL injury mechanisms, no significant differences were found regarding relativized ski length to height, side cut radius and ski dimensions (Table 2).

### Accident characteristics

Significant differences were found regarding circumstances of fall according to the four injury mechanisms (*p* < 0.001), e.g., catching the edge of the ski was more frequent the cause for forward falls with and without body rotation when compared to the backward fall with and without body rotation (75 and 67 vs. 46 and 15%) (Table 2). Skis sliding out or away and losing balance are the most frequent reported circumstances of a backward fall with (54%) and without body rotation (77%).

With regard to causes/actions when the ACL injury occurred, significant differences were found between the injury mechanisms (*p* < 0.001). However, executing a turn was the most frequent reported action in a forward fall with body rotation (56%), forward fall without body rotation (61%), in a backward fall with body rotation (68%) and in a backward fall without body rotation (39%), respectively.

### ACL injury severity

No significant differences between ACL injury mechanisms were found within the distribution of injury severity, however, a complete rupture of the ACL (66–70%) was more frequently reported than a partial tear (30–34%) among all four non-contact ACL injury mechanisms (Table 2).

## Discussion

The most important finding of this study was that ski-geometric parameters did not significantly differ between ACL injury mechanisms. The only intrinsic risk factor that showed significant differences was risk-taking behavior. Catching an edge of the ski was mostly reported for forward falls, whereas skis sliding out and loosing balance was the main cause of backward falls. In addition, most falls happened while executing a turn. Moreover, a complete rupture of the ACL was the most common type of ACL injury among all four non-contact ACL injury mechanisms.

Generally, the forward fall with body rotation (63%) was the most common self-reported ACL injury mechanism, followed by the backward fall with body rotation (25%). No significant sex difference was found within the distribution of ACL injury mechanisms, as both, females and males reported the forward fall with body rotation being the most common ACL injury mechanism. This seems to be in line with the results of other studies by Shea et al. [25], Ruedl et al. [21] and Posch et al. [15].

### Intrinsic risk factors

In accordance to the earlier study by Ruedl et al. [17], age, height, weight and skiing skill level did not significantly differ between the four ACL injury mechanisms. However, risk-taking behavior showed a significant difference between the four ACL injury mechanisms. About two-thirds of patients with an injury of the ACL due to a forward fall without body rotation reported a riskier skiing behavior, which is in contrast to the remaining three ACL mechanisms, where two-thirds of patients assessed their skiing behavior as more cautious. So far, an evidence-based explanation for this result seems not possible as age, skill level and ski geometry parameters did not differ between groups. Thus, further investigations are needed.

Only patients suffering from a forward fall without body rotation reported being mainly riskier while skiing compared to the other ACL injury mechanisms. Interestingly, catching the edge was the most common reported circumstance of fall among both, forward falls with and without body rotation. It seems that the combination of a risky skiing style and catching the edge more frequently leads to forward falls without twisting the lower extremities and the whole body.

### Ski-geometric parameters

As a novelty, the impact of ski-geometric parameters on injury mechanisms was additionally evaluated in this study. Well in accordance with a previous study by Ruedl et al. [17], relativized ski length was not significantly different between the four ACL injury mechanisms. Relativized ski length in this study with 95–97% of body height also seems to be in line with earlier results reported by Posch et al. [14], where ACL-injured skiers used skis with almost 95% of their height.

Although the sidecut radius did not significantly differ between ACL injury mechanisms, the sidecut radius seems to be somewhat smaller among forward falls with body rotation compared to the other ACL injury mechanisms. In general, the lower the sidecut radius the more a self-steering effect is given [23]. Thus, it can be potentially easier to catch the edge resulting mainly in a forward fall with body rotation [17].

Ski dimensions at the tip, waist and width of the skis were not significantly different between the four ACL injury mechanisms and, therefore, do not seem to impact different types of ACL injury mechanisms in recreational skiing. This seems somewhat surprisingly as studies found that the waist width of the ski influences kinematics, external torques and external rotation of the knee joint [29, 30]. Generally, it is recommended to use skis with a harmonious setting, reduced bending stiffness in the blade area, less sidecut and shorter length as these parameters seem to be more forgiving and, for reasons of skiing, more suitable for less experienced skiers [23].

### Accident characteristics

Well in accordance with a study by Ruedl et al. [17], catching an edge is more frequently reported for forward falls with and without body rotation (75 vs. 67%) when compared with backward falls with and without rotation (46 vs. 15%). Natri et al. [12] mentioned that losing balance is primary associated with a backward fall with body rotation, which seems to be in line with findings of the present study with 54–77% of patients suffering from a backward fall reported of losing balance prior to the accident.

Forward falls with (56%) and without body rotation (61%) and backward falls with (68%) and without rotation (39%) tend to occur more often in executing a turn than going straight and landing after a jump. These results are contrary to a study by Ruedl et al. [17], where forward falls with body rotation tend to occur more frequently in turning, when compared to all other ACL injury mechanisms (69 vs. 41%). In this study, executing a turn was the most frequent reported cause within all four ACL injury mechanisms. This seems not surprisingly as turning is the main movement on ski sloped during skiing. Potentially while executing a turn there seems to be a greater risk to lose balance and the center of pressure or catching an edge leading to a fall. The combination of the self-steering effect of the carving ski while putting the ski on the edge during turning might expose the skier to a greater risk of falling.

### ACL injury severity

Although the four ACL injury mechanisms differ regarding their biomechanical injury sequence according to Natri et al. [12], no significant differences were found between ACL injury mechanisms with regard to injury severity. However, a complete rupture of the ACL was more common than a partial tear among all injury mechanisms (66–70%). Also, Posch et al. [15] reported that 65% of injured recreational alpine skiers suffered from a complete tear of their ACL. Furthermore, Posch et al. [15] showed that among all injury mechanisms, ACL injuries with concomitant injuries occurred more frequent (53–68%) when compared to isolated ACL injuries. Compared to so-called traditional long and unshaped skis, carving skis potentially enable the skier to ski faster [24] leading to more complex injury situations where the knee joint suffers from rotational and bending movements.

When specifically focusing on the different ACL injury mechanisms, this study may provide a deeper understanding of injury causation and risk factors associated with different ACL injury mechanisms and could help to improve preventive measures especially including skiing style and skiing behavior. It is of clinical relevance to know that ski geometry does not influence ACL injury mechanisms and related injury severity.

A limitation of the study could be the selection bias, due to the specific type of patient recruitment. The sport clinic is situated close to a big ski resort and most patients admitted to or attending the sport clinic are suffering from skiing-related knee injuries. Interview days (23 per season) have been randomly selected in the same way each season to reduce the potential impact of a selection bias. However, at least with regard to the age and sex distribution our study sample seems to be well in line with the study sample of Ruedl et al. [20] and is, therefore, representative for the total skier population aged over 17 years suffering from an ACL injury in Austrian skiing regions. Furthermore, we acknowledge the possibility of recall bias especially regarding the causes and circumstances of falling leading to the ACL injury. Therefore, pictures {Natri et al. [12]} were provided of the different ACL injury mechanisms to support patients in their decision-making processes. Strengths of the study include clearly the novelty of newly investigating the impact of ski-geometric parameters on ACL injury mechanisms and second, the large number study participants when compared to the only study {Ruedl et al. [17]}, that investigated intrinsic and extrinsic risk factors between a forward twisting fall and other ACL injury mechanisms.

## Conclusion

Based on these findings, the hypothesis that ski geometry would influence the ACL injury mechanism has not been confirmed. In contrast, risk-taking behavior and accident characteristics do significantly differ between ACL injury mechanisms in recreational skiing. Thus, an individual skiing style seems to have more impact on ACL injury mechanisms than ski equipment.

## Data Availability

Data are available upon reasonable request.

## References

[1] Bahr R, Krosshaug T (2005). Understanding injury mechanisms: a key component of preventing injuries in sport. Br J Sports Med.

[2] Beynnon BD, Ettlinger CF, Johnson RJ, Hewett TE, Shultz SJ, Griffin LY (2007). Epidemiology and mechanisms of ACL injury in alpine skiing. Understanding and preventing noncontact ACL injuries.

[3] Burtscher M, Gatterer H, Flatz M, Sommersacher R, Woldrich T, Ruedl G (2008). Effects of modern ski equipment on the overall injury rate and the pattern of injury location in alpine skiing. Clin J Sport Med.

[4] Burtscher M, Sommersacher R, Ruedl G, Nachbauer W, Johnson RJ, Shealy JE, Langran M (2009). Potential risk factors for knee injuries in alpine skiers. Skiing trauma and safety.

[5] Cusimano MD, Kwok J (2010). The effectiveness of helmet wear in skiers and snowboarders: systematic review. Br J Sports Med.

[6] Ekeland A, Rødven A, Johnson RJ, Shealy JE, Senner V (2011). Skiing and boarding injuries on Norwegian slopes during two winter seasons. Skiing trauma and safety.

[7] Ekeland A, Rødven A, Johnson RJ, Shealy JE, Greenwald RM, Scher IS (2012). Injuries in alpine skiing, telemarking, snowboarding and skiboarding related to gender and ability. Skiing trauma and safety.

[8] Hewett TE, Myer GD, Ford KR (2006). Anterior cruciate ligament injuries in female athletes: part 1, mechanism and risk factors. Am J Sports Med.

[9] Kim S, Endres NK, Johnson RJ, Ettlinger CF, Shealy JE (2012). Snowboarding injuries. Trends over time and comparisons with alpine skiing injuries. Am J Sports Med.

[10] LaPorte JD, Binet MH, Constans D, Johnson RJ, Zucco P, Shealy JE (2000). Evolution of ACL ruptures in French ski resorts 1992–1999. Skiing trauma and safety.

[11] Majewski M, Susanne H, Klaus S (2006). Epidemiology of athletic knee injuries: a 10-year study. Knee.

[12] Natri A, Beynnon BD, Ettlinger CF, Johnson RJ, Shealy JE (1999). Alpine ski bindings and injuries. Current findings Sports Med.

[13] Panisset JC, Gonzalez JF, de Lavigne C, Ode Q, Dejour D, Ehlinger M (2019). ACL reconstruction in over-50 year-olds: comparative study between prospective series of over-50 year-old and under-40 year-old patients. Orthop Traumatol Sur.

[14] Posch M, Ruedl G, Schranz A, Tecklenburg K, Burtscher M (2019). Is ski boot sole abrasion a potential ACL injury risk factor for male and female recreational skiers?. Scand J Med Sci Sports.

[15] Posch M, Schranz A, Lener M, Tecklenburg K, Burtscher M, Ruedl G (2020). In recreational alpine skiing, the ACL is predominantly injured in all knee injuries needing ospitalization. Knee Surg Sports Traumatol Arthrosc.

[16] Razi M, Soufali AP, Ziabari EZ, Dadgostar H, Askari A, Arasteh P (2021). Treatment of concomitant ACL and MCL injuries: spontaneous healing of complete ACL and MCL tears. J Knee Surg.

[17] Ruedl G, Linortner I, Schranz A, Fink C, Schindelwig K, Nachbauer W (2009). Distribution of injury mechanisms and related factors in ACL-injured female carving skiers. Knee Surg Sports Traumatol Arthrosc.

[18] Ruedl G, Webhofer M, Linortner I, Schranz A, Fink C, Patterson C (2011). ACL injury mechanisms and related factors in male and female carving skiers: a retrospective study. Int J Sports Med.

[19] Ruedl G, Webhofer M, Helle K, Strobl M, Schranz A, Fink C (2012). Leg dominance is a risk factor for noncontact anterior cruciate ligament injuries in female recreational skiers. Am J Sports Med.

[20] Ruedl G, Philippe M, Sommersacher R, Dünnwald T, Kopp M, Burtscher M (2014). Current incidence of accidents on Austrian ski slopes. Sportverletz Sportschaden.

[21] Ruedl G, Helle K, Tecklenburg K, Schranz A, Fink C, Burtscher M (2016). Factors associated with self-reported failure of binding release among ACL injured male and female recreational skiers: a catalyst to change ISO binding standards?. Br J Sports Med.

[22] Russel K, Christie J, Hagel BE (2010). The effects of helmets on the risk of head and neck injuries among skiers and snowboarders: a meta-analysis. Can Med Assoc J.

[23] Senner V, Michel FI, Lehner S, Brügger O (2013). Technical possibilities for optimising the ski-binding-boot functional unit to reduce knee injuries in recreational alpine skiing. Sports Eng.

[24] Shealy JE, Ettlinger CF, Johnson RJ (2005). How fast do winter sports participants travel on alpine slopes?. J ASTM Intl.

[25] Shea KG, Archibald-Seiffer N, Murdock E, Grimm NL, Jacobs JC, Willick S (2014). Knee injuries in downhill skiers: a 6-year survey study. Orthop J Sports Med.

[26] Shi H, Jiang Y, Ren S, Hu X, Huang H, Ao Y (2020). Sex differences in the knee orthopaedic injury patterns among recreational alpine skiers. BMC Sports Sci Med Rehabil.

[27] Steadman JR, Matheny LM, Briggs KK, Rodkey WG, Carreira DS (2012). Outcomes following healing response in older, active patients: a primary anterior cruciate ligament repair technique. J Knee Surg.

[28] Sulheim S, Ekeland A, Bahr R (2007). Self-estimation of ability among skiers and snowboarders in alpine skiing resorts. Knee Surg Sports Traumatol Arthrosc.

[29] Zorko M, Nemec B, Babič J, Lesnik B, Supej M (2015). The waist width of skis influences the kinematics of the knee joint in alpine skiing. J Sports Sci Med.

[30] Zorko M, Hirsch K, Šarabon N, Supej M (2020). The influence of ski waist-width and fatigue on knee-joint stability and skier’s balance. J Appl Sci.

